# The interruption of *Onchocerca volvulus* and *Wuchereria bancrofti* transmission by integrated chemotherapy in the Obongi focus, North Western Uganda

**DOI:** 10.1371/journal.pone.0189306

**Published:** 2017-12-18

**Authors:** Lakwo Thomson Luroni, Matwale Gabriel, Edridah Tukahebwa, Ambrose Winston Onapa, Benjamin Tinkitina, Ephraim Tukesiga, Michael Nyaraga, Anna Mary Auma, Peace Habomugisha, Edson Byamukama, David Oguttu, Moses Katabarwa, Thomas Raymond Unnasch

**Affiliations:** 1 Vector Control Division, Ministry of Health, Kampala, Uganda; 2 Envision/RTI- Neglected Tropical Diseases Control Programme, Kampala, Uganda; 3 Kabarole District Local Government, Fort Portal, Uganda; 4 Moyo District Local Government, Medical Department, Moyo, Uganda; 5 The Carter Center, Uganda office, Kampala, Uganda; 6 The Carter Center, Atlanta, United States of America; 7 University of South Florida, Global Health Infectious Disease Research, College of Public Health, Tampa, FL, United States of America; New England Biolabs Inc, UNITED STATES

## Abstract

**Intervention:**

Few studies have documented the interruption of onchocerciasis and Lymphatic Filariasis (LF) by integrated chemotherapy in Uganda. The study describes the interruption of transmission of the two diseases co-endemic in Obongi focus, north western Uganda. Base line data for Onchocerciasis and LF were collected in 1994 and 2006, respectively. Annual mass drug administration for onchocerciasis (Ivermectin) and Lymphatic Filariasis (Ivermectin + albendazole) was conducted for 20 and 6 years, respectively. Thereafter, assessments by skin snip, larval searches in rivers and human landing catches were performed. Children <10 years were screened for IgG4 antibodies using Ov16 ELISA technique in 2013. LF Pre-TAS and TAS1 were conducted in sentinel sites. ITN coverage and utilization for the implementation unit was also reported.

**Intervention coverage:**

Onchocerciasis treatment coverage was <80% but improved with the introduction of CDTI in 1999. While for LF, effective coverage of >65% was achieved in the six treatment rounds. Household ownership of ITN’s and utilization was 96% and 72.4%., respectively.

**Impact:**

Parasitological examinations conducted for onchocerciasis among 807 adults and children, revealed a reduction in mf prevalence from 58% in 1994 to 0% in 2012. Entomological monitoring conducted at the two sites had no single *Simulium damnosum* fly caught. Serological analysis using Ov16 ELISA for onchocerciasis revealed that out of the 3,308 children <10 years old screened in 2013, only 3/3308 (0.091%) positive cases were detected. All Ov16 positive children were negative when tested for patent infection by skin snip PCR. A reduction in LF microfilaria prevalence from 2.5% (n = 13/522) in 2006 to 0.0% (n = 602) in 2014 was observed. LF TAS1 conducted in 2015 among 1,532 children 6–7 years, all were negative for antigens of *W*. *bancrofti*.

**Conclusion:**

The results concluded that interruption of onchocerciasis and LF has been achieved.

## Introduction

Elimination of neglected tropical diseases (NTDs) has recently emerged on the global health agenda and gained prominence with the release of the global plan to combat NTDs by the World Health Organization [1]. Onchocerciasis (river blindness) is caused by *Onchocerca volvulus* (Nematoda: Filaroidea) which is transmitted by blackflies (Diptera: Simuliidae) and has been targeted for elimination of transmission (which will also result in elimination of the parasite). Common symptoms include severe itching, skin lesions, vision impairment (including blindness) and epilepsy [2]. Onchocerciasis is endemic in parts of Africa, Latin America, and Yemen, but over 99% of cases are found in sub-Saharan Africa [3]. Historically, the disease has been a serious public health problem in sub-Saharan Africa and hindered socioeconomic development in the affected areas [4], but the introduction of Community Directed Treatment with Ivermectin (CDTI) in 1996 to control the disease has reduced the burden of disease to such an extent that it is no longer a public health problem in most endemic areas [5]. Later, studies in Mali and Senegal have proved the feasibility of onchocerciasis elimination through ivermectin treatment in some hyper-endemic foci in West Africa [6]. This has provided the momentum and arguments for a shift in the strategic goal from control to elimination in Africa.

In Uganda, an onchocerciasis elimination policy was launched in 2007 using CDTI often supplemented by vector control/elimination in 14 districts in six foci and in some areas the disease co-existed with Lymphatic Filariasis [7]. There has been steady progress in the elimination efforts and recognizable achievements have been made in a number of foci where onchocerciasis transmission has been interrupted [8–10]. The country is pursuing a focus by focus elimination approach based on the national and WHO guidelines [11].

Lymphatic Filariasis (LF) is caused by infection with *Wuchereria bancrofti* (Nematoda: Filaroidea) transmitted by mosquitoes (Diptera: Culicidae) (*Anopheles gambiae*, *An*. *funestus)* in Uganda. The main signs and symptoms of LF are mainly characterized by lymphodema and hydrocele [12].The Programme to Eliminate Lymphatic Filariasis was launched in the greater Lira and Katakwi districts in 2002. There was a gradual scale up until all the 54 endemic districts were under MDA by 2010. The elimination strategy for LF is normally based on mf reservoir reduction and morbidity control [13], however, for the case of Uganda only the former is being prioritized. The population at risk receives medicines (ivermectin and albendazole) through mass drug administration in all the implementation units (endemic districts). Impact assessments for LF were conducted in sentinel and spot check sites to ascertain the microfilaria/antigenemia prevalence in the affected population. In an effort to determine the end game, Transmission Assessment Surveys (TAS) was conducted in eligible evaluation units using standard procedures; and MDA was halted based on the TAS results [14]. Chemotherapy (by Mass Drug Administration to whole communities) has been the main strategy in the elimination of neglected tropical diseases including LF [15] and Togo is one of the countries in Africa that has successfully eliminated LF through this approach [16]. However, the roles of malaria vector control through Insecticide-Treated bed Nets (ITNs) and Indoor Residual Spraying (IRS) have been less reported, although they undoubtedly play an important role in the reduction of the burden of LF where it is transmitted by *Anopheles* If achievements made in LF are to be sustained in most African countries including Uganda, integrated vector management (IVM) is crucial and this has been emphasized in a number of studies for combating vector borne disease transmission [17]. In this paper, we describe the simultaneous interruption of transmission of both onchocerciasis and lymphatic filariasis in Obongi focus, where they were co-endemic.

## Materials and methods

### Ethics statement

This protocol was reviewed and approved by the Vector Control Division, Research Ethics Committee of the Ministry of Health. Approval was obtained from relevant local authorities before accessing the communities. Written informed consent was obtained from participants above 18 years, while those in the age group of 5–17 years, their parents or guidance were consented through administration of assent forms as described in section 5.0 of the guidelines provided by the Uganda National council for Science and Technology [18].

### Study area

The study area was the Obongi focus located in Moyo District, north western Uganda. Obongi focus is slightly smaller than Moyo District with population at risk of 38,665 constituting only 0.6% of the total population at risk. It is constituted by the Aliba, Itula and Gimara sub-counties in Moyo District. The River Nile forms a natural boundary to the east, while in the west is Yumbe district and south is Arua district. Annual ivermectin treatment started in this focus in 1993 among the population whose occupation is mostly subsistence farming supplemented by fishing. The isolation of Obongi focus from Madi mid-north focus is by the R. Nile. However, the West Nile focus that lies to the south is more than 50 km from the Obongi focus. Comparatively, onchocerciasis is more focal in the region whereas LF is endemic in the whole district.

### Base line data collection

#### Onchocerciasis

In 1993 Epidemiological survey was conducted in one of the sentinel villages located in the focus to establish the magnitude of the disease prior administration of ivermectin. A total of 160 people from Nyimanji camp were clinically and parasitologically examined following the standard procedures [19].

#### Lymphatic filariasis

Moyo district where Obongi focus is situated has one sentinel site in Dufile village. A total of 522 inhabitants were mobilized for this survey and were requested to assemble at a central place from 9:00 pm. Blood sample were collected in a capillary tube, and this was used to prepare blood slide with three lines. The slides were heamoglobinized, dried and fixed with alcohol (methanol). The slides were stained in Giemsa and later examined for microfilaria using standard procedures [20].

### Annual mass treatment with ivermectin and albendazole

Annual mass treatment with ivermectin in Obongi focus began in 1993 by the River Blindness Foundation (RBF), however, later on The Carter Center took over the responsibilities of RBF in Uganda. APOC assistance for community-directed treatment with ivermectin (CDTI) activities in this focus commenced in 1999 and this covered all 61 communities in the three sub-counties. The role of the Ministry of Health since then has been coordination, technical guidance to districts, guidelines and standards on onchocerciasis and supervisory roles in endemic districts. In 2006, treatment for LF was extended to this focus with the support of The Carter Center and later by Envision/RTI. Treatment for Onchocerciasis was on an annual basis from 1993–2014, conducted in the months of April in an integrated manner through the program by adding Albendazole during the treatment. The data for all treatments was captured in the NTD integrated register and all reporting related to MDA in this focus followed what has been stipulated in the national Ministry of Health NTD Master Plan.

### Onchocerciasis assessments

#### Entomological monitoring in the focus

Blackfly catching points were established along R. Omvoso in the Obongi focus. Two vector collectors were recruited and trained on human landing catches for *Simulium* and catches was conducted twice a week for 11-hours (07:00–18:00) from April 2012 to July 2013 using the same procedures previously described [21].

#### Clinical and parasitological assessment

Rapid assessment to establish the presence of palpable nodules was conducted in Obongi focus in 2012. Adults and children were skin snipped from eight villages in the three sub-counties of Gimara, Aliba and Itula. The standard protocol for skin snipping was followed, whereby two skin snips were taken from the posterior iliac crest for every selected adult. The skin snips were read under low (x40) magnification after 12-hour incubation at ambient temperature in normal saline solution [22].

#### Onchocerciasis serology assessment

Serological assays testing for *Onchocerca volvulus* exposure was conducted among children (4-<10 years) resident in Obongi focus in July 2011. Children (n = 3308) were selected by a multi-stage stratified sampling scheme applied at the parish administrative level. Parishes were chosen for sampling using a simple random sampling scheme. In every selected parish, all children under 10 years had equal chances of being selected. The minimum number of children sampled from each parish was calculated based upon the percentage of overall population of the focus that resided in each parish [23]. Blood spot collection was by finger prick from each child enrolled in the study. The blood samples were air dried and transported to the laboratory in zip-lock bags. Two 6-mm punches of saturated filter paper were placed in phosphate buffered saline (PBS)-Tween 0.05% and bovine serum albumin (BSA) 5% buffer and eluted overnight at 4°C. The elution was run in duplicate in a standard enzyme linked immunosorbent assay ELISA to detect IgG4 antibodies against the OV 16 recombinant antigen following standard procedures [24]. Putative positive samples were re-tested with plates coated with Ov16–GST and with control GST. Samples that gave a positive reading in both Ov16 assays and were negative for GST alone were scored as confirmed negative. The 95% CIs for collections in which positive serum samples were identified were calculated as previously described [25].

#### LF transmission follow up assessment survey

LF country mapping was conducted in 2001–2003 using the Circulating Filariasis Antigen rapid cards (AMRAD or BINAX) [26]. In Moyo, Dufile and Aliba were the two sites randomly selected because they had registered *Wuchereria bancrofti* antigenemia of 20.3% and 8.8% respectively (see S1 Table). Following the baseline data collection, mass treatment with Ivermectin and Albendazole was initiated in 2006. MDA impact assessment surveys were conducted in Moyo district as an implementation unit in 2011 and 2014. In 2015, through the recommendation of Regional Programme Review Group (RPRG), TAS was conducted in Moyo district. The sampling frame was communities located in Moyo district where 6-7year olds were targeted. A cluster sampling design using survey sampling builder was used [27]. The targeted number of communities or enumeration areas was 42 with targeted sample size of 1,532. A total of 1532 children were screened using ICT diagnostic test to detect antibodies for *W*. *bancrofti* [28].

### Malaria intervention in Moyo district

Lymphatic filariasis and malaria have the same vector, *Anopheles gambiae* and *A*. *funestus*. This makes the two diseases to benefit from each other in terms of interventions targeting the vector. In order to obtain information related to use of long lasting insecticide treated nets (ITNs), Uganda Malaria indicator survey report for 2014–15 was consulted. This document presents national and regional estimates of a range of malaria indicators and thus provides a robust and comprehensive picture of malaria control in Uganda. It captures both biological and behavioural information relevant to malaria and provides a useful reference tool and evidence base for national policy decision making [29].

## Results

### Base line data collection

#### Onchocerciasis

The prevalence of microfilaria was 58% among the 160 inhabitants examined from Nyimanji camp. The mfs/mg was 9.67 and geometric mean 2.60. This prevalence along with the nodules prevalence categorized the focus as a meso-endemic area and was eligible for mass drug administration with Ivermectin.

#### Lymphatic filariasis

In 2006, the microfilaria prevalence recorded among the population examined from Dufile village was 2.5% (13/522), This was the figure that enabled the mass drug administration with ivermectin and albendazole to be initiated in the whole district as an implementation unit.

### Annual mass treatment with ivermectin and albendazole

**Fig 1:**
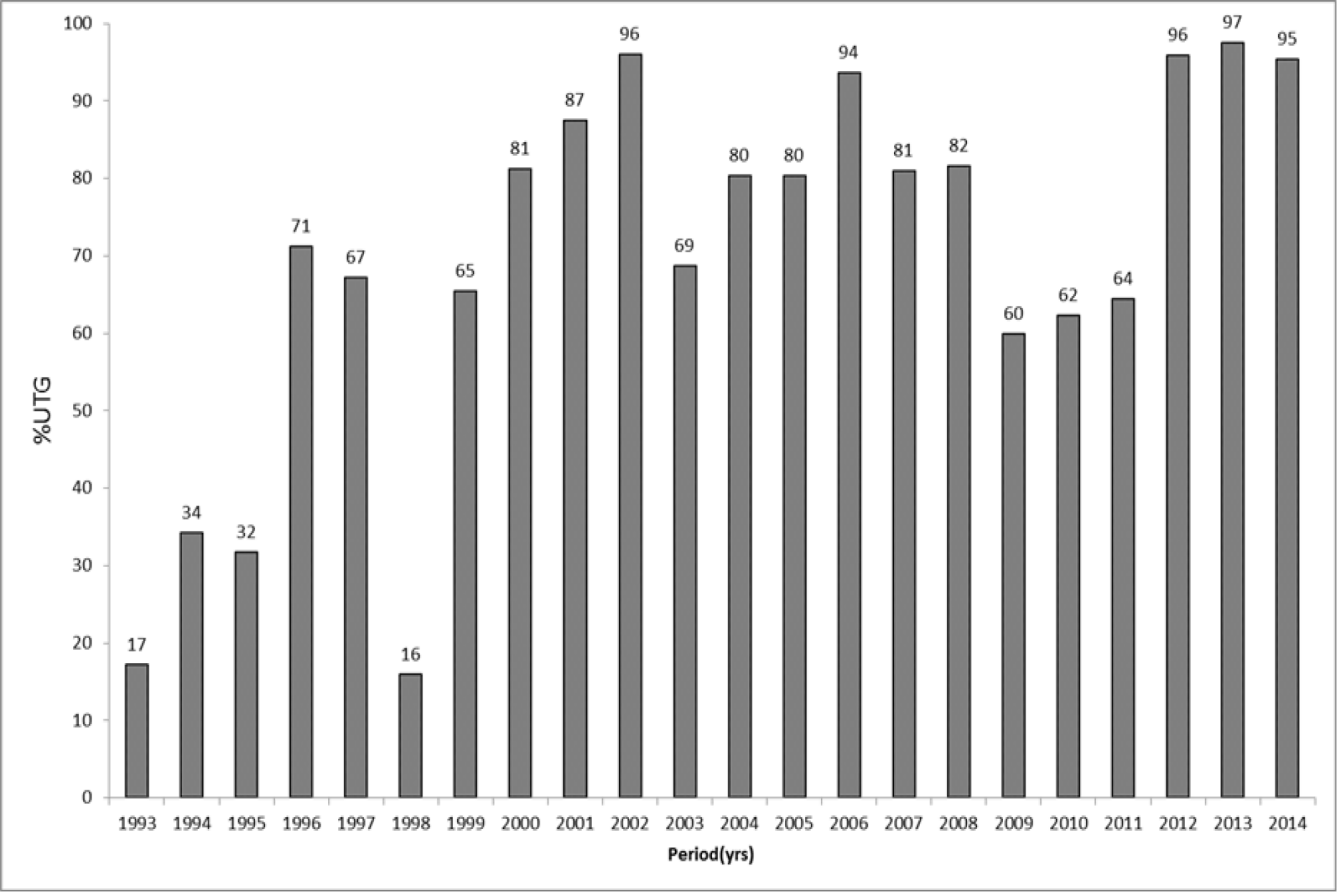
History of treatment coverage with ivermectin from 1993–2014 in Obongi onchocerciasis focus.

**Fig 2:**
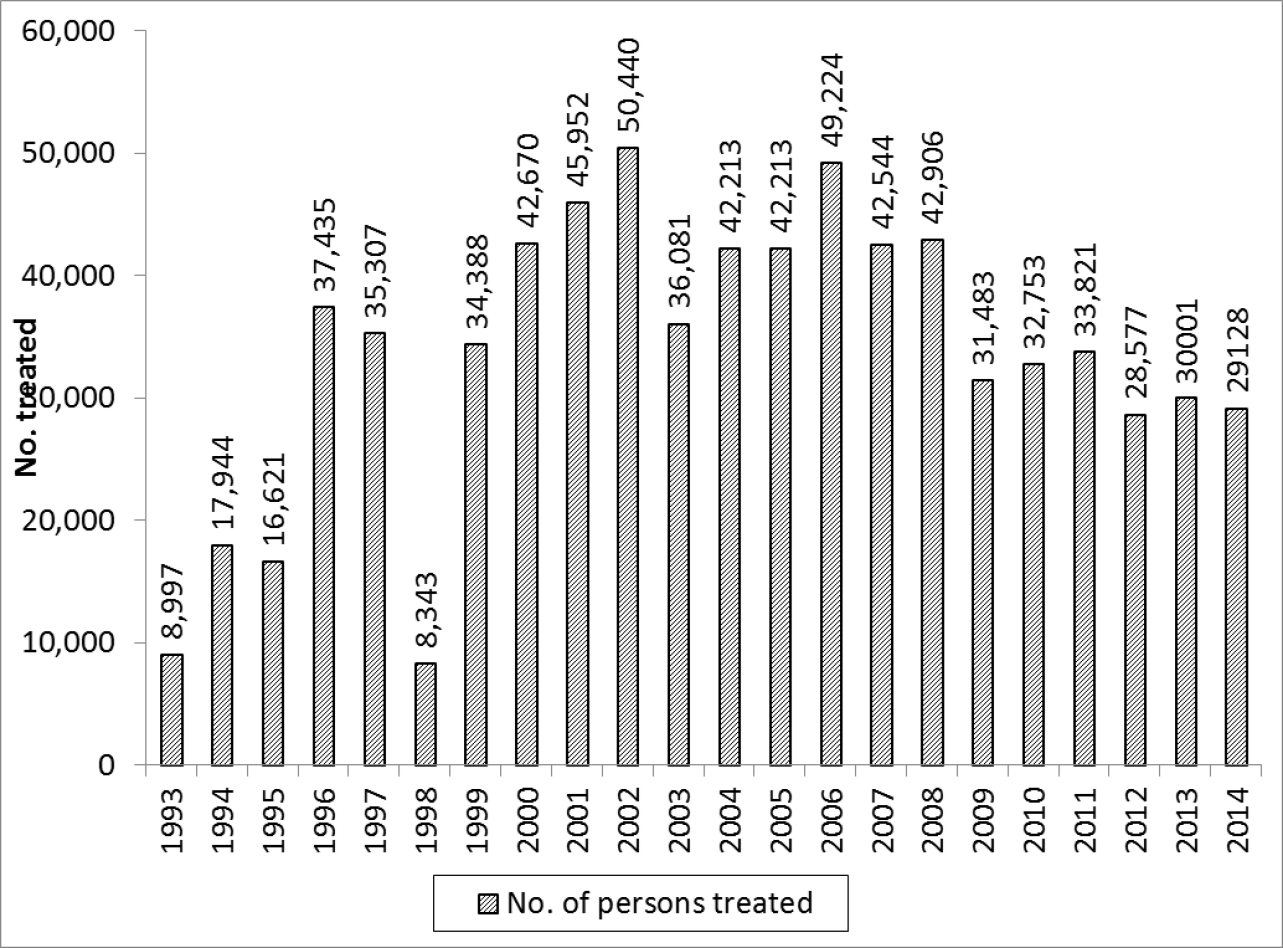
Number of people treated with ivermectin from 1993–2014 in Obongi onchocerciasis focus.

The results of Ivermectin mass treatment for onchocerciasis in Obongi focus is shown in “Figs 1 and 2”. Low treatment was observed from 1993–1995 when the programme began and drastically reduced to 16% in 1998. There was gradual increase in treatment coverage from 1999 until 2009 when there was a slight drop to <65% (“Fig 1”). The number of persons treated in this focus has been fluctuating during the 20 years and also influx of refugees from South Sudan; with the lowest population of 8,343 in 1998 and the highest of 50,440 in 2002. By the time when treatment was halted in 2014 the number of people being treated per round was 29,128 (“Fig 2”).

**Fig 3:**
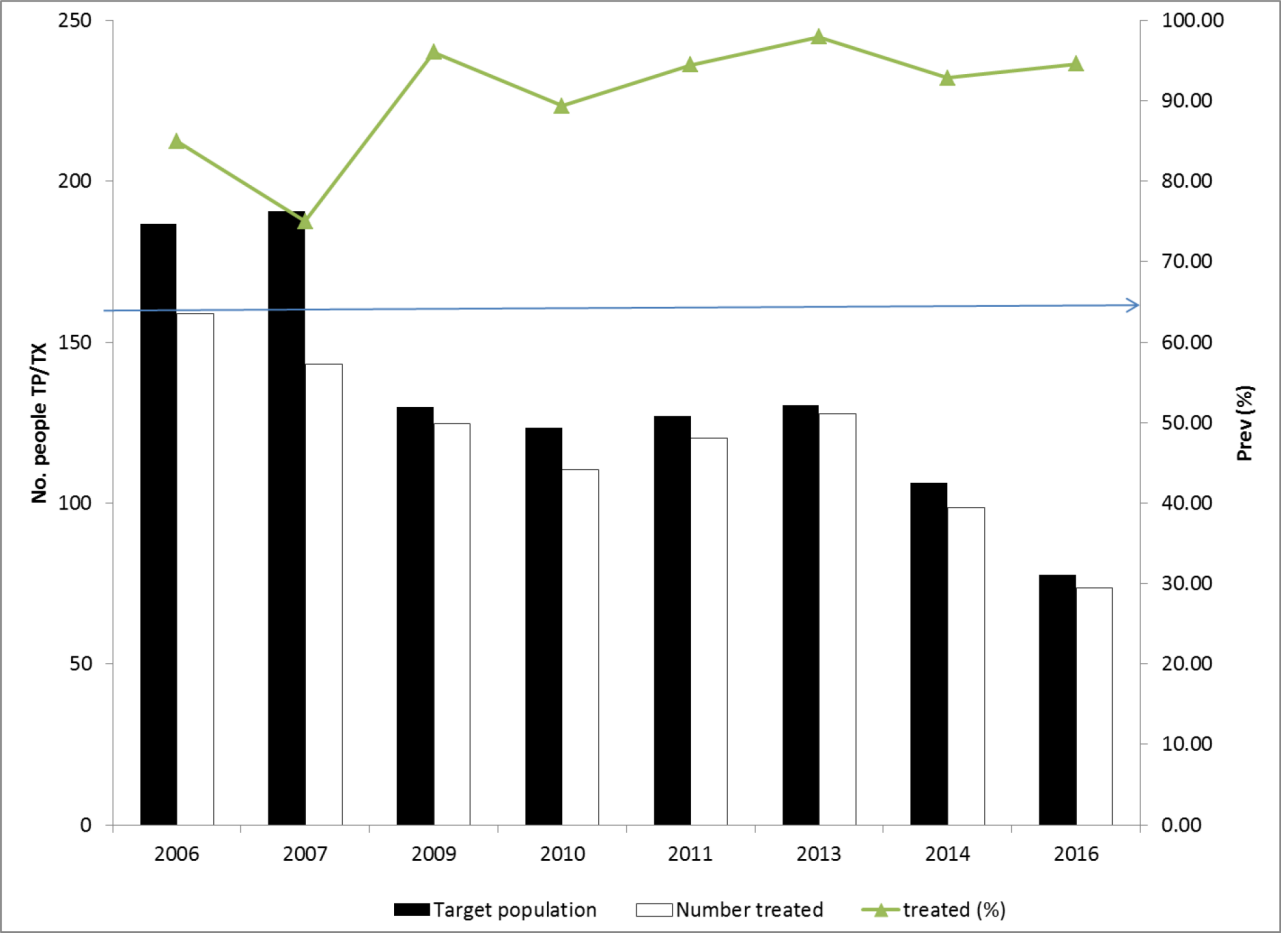
Percent treatment coverage for lymphatic filariasis from 2006–2016 in Moyo districts.

The LF mass treatment with Ivermectin and Albendazole started in 2006 and all the six treatment rounds had been effective above 70% coverage. Mass treatment was not conducted in 2015 due to fact that TAS was being conducted in the district (“Fig 3”).

### Clinical and parasitological assessment for onchocerciasis

A total of 807 adults and children were clinically and parasitological examined. Only two adults (0.2%) had nodules from only one village, while among the children, none had nodules. Parasitological examination by skin snip found only one adult (0.1%) positive with microfilariae. No child was found with microfilaria (“Table 1”).

**Table 1:** Microfilariae and nodule prevalence in Obongi focus, northwestern Uganda in 2012.

Village	Adults					Children		
	Adults Examined	No. Positive with mf	% mf	No. Positive with Nod	% Nod	No. Examined	No. Positive with mf	% mf
Maduga	110	0	0.0	0	0.0	3	0	0
Lionga	128	0	0.0	0	0.0	15	0	0
Liwa South	123	0	0.0	0	0.0	18	0	0
Yakinemiji*	71	1	1.4	2	2.8	34	0	0
Dilokata	90	0	0.0	0	0.0	9	0	0
Alibabito	137	0	0.0	0	0.0	8	0	0
Indilinga	108	0	0.0	0	0.0	0	0	0
Angaliacini	40	0	0.0	0	0.0	19	0	0
**TOTAL**	**807**	**1**	**0.1**	**2**	**0.2**	**106**	**0**	**0**

* Follow up of the above mf positive cases indicated that they were immigrants from one of the endemic districts in the focus (Kitgum)

### Entomological monitoring for onchocerciasis

In adult fly catches conducted at Kochi Boma and Lomunga catching sites from 2012 until 2016 no *Simulium damnosum* flies were collected over a total of 480 collection days (“Table 2”). Larval searches conducted on two river systems (R. Omvoso, R. Nyawa) did not encounter any immature stages of *S*. *damnosum* in the trailing vegetation or substrates, and there were also no freshwater crabs caught during the prospections.

**Table 2:** Human landing catches at Kochi Boma and Lomunga sites in Obongi focus, northwestern Uganda.

Year	Catching sites	
	Kochi Boma	Lomunga
2012	0	0
2013	0	0
2014	0	0
2015	0	0
2016	0	0
**Total**	**0**	**0**

### Serological Assessment for Onchocerciasis

The results of serological analysis using Ov16 ELISA is shown in ‘Table 3” Out of the 3,308 children <10 years old screened in 2013, three positive cases (3/3308: 0.09%) were detected. However, the three serologically positive children were subjected to confirmatory skin snip O-150 PCR assays following current WHO guidelines. All the three positive children were found to be negative in the confirmatory assay.

**Table 3:** Showing results of children <10 years screened with Ov16 ELISA in Obongi focus.

Age group	Number screened	Positive	% Positive	95% CI (%)	
				Lower Limit	Upper Limit
1–4	1,813	2	0.110	0.008	0.31
5–9	1,495	1	0.067	0.0001	0.26
**Total**	**3,308**	**3**	**0.091**	**0.015**	**0.22**

### Parasitological and transmission assessment surveys for LF

There was gradual reduction in the microfilaria prevalence from 2.5% (n = 522) at baseline in 2006 to 0.8% (n = 654) in 2011 and 0.0% (602) in 2014 based on night blood for assessing *Wuchereria bancrofti* microfilaria. In 2015, LF TAS conducted among 1532 children 6–7 years in this focus was all negative (0.0%) for antibodies of *W*. *bancrofti* (“Table 4”; S2 Table).

**Table 4:** Impact assessments of lymphatic filariasis (elephantiasis) from 2006 to 2015 in Moyo district.

*Sub-county	**Year**	**Number examined**	**No. infected**	**% infected**	**95% CI**	
					**Lower Limit**	**Upper Limit**
Dufile	2006 –Base-line	522	13	2.490	0.0144	0.0431
Dufile/Aliba	2011 –follow up	654	5	0.874	0.0010	0.0143
Dufile/Itula	2014 –follow up	602	0	0.000	0.000	0.0000
**Lymphatic filariasis transmission Assessment survey (TAS) in children age 6–7 years in Moyo district.**						
**District**	**Year**	**No. Examined**	**Total infected**	**% infected**	**95% CI**	
					**Lower Limit**	**Lower Limit**
Moyo	2015	1532	0	0	0.000	0.000

*Obongi sub-county was a spot check site for LF.

## Discussion

The results from the impact assessment surveys for onchocerciasis and LF strongly suggest that the transmission of both diseases have been interrupted. These achievements were associated with the effective MDA treatment rounds against both onchocerciasis and LF. However, at the same time the onchocerciasis vector seems to have disappeared, and either the MDA or the vector disappearance could have eliminated transmission, but the effect of both operating together might have accelerated the elimination of onchocerciasis. The challenge the programme experienced was the fluctuation in the population figures and treatment coverage due to human population movement attributed to influx of refugees because of insecurity outside the focus, (Lord’s Resistance Army (LRA) and South Sudan conflict at that time. Despite this, the figures for onchocerciasis treatment stabilized following the introduction of CDTI in 1999 where the communities were empowered to take responsibility for the health of their communities. CDTI strategy provided a soft landing for the LF programme in 2006 because the population was already aware of mass treatment strategy. The impact of MDA in reducing prevalence levels of *W*. *bancrofti* has been well documented [30], with a combination of ivermectin and albendazole appearing to give a faster but shorter-lived reduction in microfilaria rates than DEC and albendazole [31]. However, treatment compliance in LF still remains a challenge in many African countries [32]; obtaining effective treatment coverage in this focus demonstrated the effectiveness of health education and social mobilization activities conducted by the programme.

The clinical and parasitological assessments for onchocerciasis demonstrated that mass treatment with ivermectin contributed to a great reduction in the burden of the disease. In Obongi focus it was evident that annual treatment with ivermectin could have attributed to the interruption of transmission. In this focus, there was only one village where microfilaria positive case was encountered and follow-up revealed that this person was from Madi-mid north focus, an area where there is still ongoing transmission. The proximity of Moyo district to south Sudan makes its border porous, and due to the over two decades of LRA armed conflict there had been an inevitable influx of population movement across the borders. Cross-border movements have been recognized as one of the factors fueling transmission of onchocerciasis and other NTDs in the Africa region and present a big threat to the elimination effort [33]. The need for countries to strengthen cross border collaborations becomes very crucial if the goal of eliminating onchocerciasis by 2025 is to be achieved in Africa.

In the Obongi focus, the main vector has been reported as *S*. *damnosum* s.l. [34,35]. However, the absence of the vector during the 4 years of monitoring could possibly be attributed to some ecological changes in the area. It is hypothesized that environmental changes in the ecology of the river systems in the focus could have probably resulted into the disappearance of the vector. For instance, in Wadelai focus in the same region *S*. *damnosum s*.*l*. vector also disappeared without any vector control measures [36]. It is not very certain when the vector disappeared but its absence in the focus indicates that recrudescence of onchocerciasis is extremely unlikely.

Ov16 ELISA serology has been recommended by WHO as one of the diagnostic test to determine end-point in the interruption of onchocerciasis transmission. In this focus, the parasitological and entomological indicators were below the threshold recommended for interruption of transmission. According to national and WHO guidelines, once the threshold prevalence of 95% confidence level below 0.1% has been achieved, a focus is re-classified as transmission interrupted. The guidelines recommend that any individual found to be positive in the serological assay be subjected to a confirmatory test for patent infection by PCR skin snip analysis. In the original serological survey, three children were confirmed positive. However, all three were negative in the confirmatory PCR skin snip. The calculated point prevalence for patent infection in the children tested was thus 0.0%, with an upper bound of the 95% interval of 0.06%. This satisfies the metric put forth by the guidelines, supporting the entomological observations that transmission is no longer occurring in this focus.

The magnitude of Lymphatic filariasis in Uganda was reported to be high at the beginning of the programme in 2002. The approach to transmission control was through treating the affected population with ivermectin and albendazole. The supplementary intervention has been the use of insecticide treated mosquito nets and indoor residual spraying in households supported by the Malaria Control Programme. It was however, noted that the ITN coverage and utilization in the region was quite high and this could have been one of the factors that accelerated the elimination of LF in this implementation unit. Due to the phased mapping approach in LF, intervention was initiated in this IU in 2006 and this benefitted from the existing medicine delivery system that Onchocerciasis control programme had established in the community. The impact assessment results portrayed in 2014 could have been attributed to a combination of ITN’s and the effective mass treatment for LF in the focus. But nevertheless, the implementation of six effective treatment rounds of ivermectin and albendazole could have provided additional boost in interruption of transmission, yet World Health Organization only recommends five effective rounds. Despite the challenges of population movement within the district and across borders, implementations of interventions were kept on course, yet in other countries within the region the challenge of poor compliance has been reported [37]. The scale up of malaria vector control in Africa has been promising and has increased significantly since 2005, with a three-fold increase in ITN ownership and indoor residual spraying (IRS) coverage. However, coverage varied dramatically across countries; some regions reported >70% ITNs ownership and regular IRS activity, while others had no coverage in remote rural populations where the risk of LF was potentially high and co-endemic with high risk Loa loa [38]. In Uganda, a marked reduction in *W*. *bancrofti* infection and infectivity in humans was observed in one of the LF districts, where both MDA and LLINs were used to reduce transmission [39]. In the current study, ITN coverage and utilization were reported to be 96% and 72.4% respectively; these figures are quite high to have an impact on the burden of LF. However, there is need for further studies to investigate the impact of vector control on anopheline-transmitted LF in an endemic area not benefitting from MDA and this would be valuable to determine the effect of such interventions. The negative ICT test observed among children 6–7 years in 2015 still further confirm the interruption of LF transmission in the focus. TAS using ICT among these school children has been used in Uganda and elsewhere to determine the circulating filarial antigens and break point for LF transmission [40].

The co-endemicity of Onchocerciasis and Lymphatic filariasis in this focus will provide the first experience of managing post-treatment surveillance. Although the surveillance for the two diseases has different guidelines, planning for their surveillance needs to consider the aspects of integration. Diagnostic tools like Ov16 and Wb123 serological tests have been developed as individual, standalone, rapid (10- to 20-min) lateral flow tests for point-of-contact detection of *O*. *volvulus*-specific or *W*. *bancrofti*-specific IgG4 antibodies [41, 42]. These tests can be used with serum, whole blood, or eluates of dried blood spots and have been shown to detect specific antibodies in individuals with infections that span many geographically diverse regions of the world where filarial infection is endemic. In the Obongi focus, this diagnostic test can be very useful for post treatment surveillance and determining the endgame of the two diseases. Component of health education and sensitization for the two diseases can be integrated during PTS since all of them have been using the same health education materials developed by the NTD programme.

## Conclusion

This study has documented the decline and interruption of transmission of *Onchocerca volvulus* and *W*. *bancrofti* infection in a previously Onchocerciasis and LF co-endemic area of Uganda. The interruption of transmission now protects a population of 34,181 people against onchocerciasis while for LF the whole district is protected from the risk of contracting the disease. However, the need to institute a strong surveillance system to guard against recrudescence or re-invasion by the parasites cannot be overemphasized.

## Supporting information

S1 Table CFA Grand table for Uganda. The data shows the two sites of Dufile and Aliba in Moyo district including altitude, coordinates and CFA results.
(XLS)

S2 Table TAS results for Moyo evaluation unit. Shows the number of villages assessed in Moyo EU, number screened and the ICT results.
(XLSX)

S3 Table TAS eligibility, design and results for Moyo district, 2014. Shows the data for sentinel and spot sites for EU, survey design using sampling builder and results from ICT screening.
(XLSX)
